# Increased abundance of ADAM9 transcripts in the blood is associated with tissue damage

**DOI:** 10.12688/f1000research.6241.2

**Published:** 2016-10-24

**Authors:** Darawan Rinchai, Chidchamai Kewcharoenwong, Bianca Kessler, Ganjana Lertmemongkolchai, Damien Chaussabel

**Affiliations:** 1 Systems Biology Department, Sidra Medical and Research Center, Doha, Qatar; 2 Cellular and Molecular Immunology Unit, The Centre for Research and Development of Medical Diagnostic Laboratories (CMDL), Faculty of Associated Medical Sciences, Khon Kaen University, Khon Kaen, 40000, Thailand

**Keywords:** ADAM9, Data mining, Transcriptomics, RNAseq, Microarray

## Abstract

**Background: **Members of the ADAM (a disintegrin and metalloprotease domain) family have emerged as critical regulators of cell-cell signaling during development and homeostasis. ADAM9 is consistently overexpressed in various human cancers, and has been shown to play an important role in tumorigenesis. However, little is known about the involvement of ADAM9 during immune-mediated processes.

**Results: **Mining of an extensive compendium of transcriptomic datasets identified important gaps in knowledge regarding the possible role of ADAM9 in immunological homeostasis and inflammation: 1) The abundance of ADAM9 transcripts in the blood was increased in patients with acute infection but, 2) changed very little after *in vitro* exposure to a wide range of pathogen-associated molecular patterns (PAMPs). 3) Furthermore it was found to increase significantly in subjects as a result of tissue injury or tissue remodeling, in absence of infectious processes.

**Conclusions: **Our findings indicate that ADAM9 may constitute a valuable biomarker for the assessment of tissue damage, especially in clinical situations where other inflammatory markers are confounded by infectious processes.

## Introduction

Over the recent years “deep” molecular phenotyping technologies have become widely available to biomedical researchers. As a consequence collections of large-scale datasets held in public repositories are rapidly expending. For instance, GEO, the NCBI Gene Expression Omnibus, is comprised of over 70,000 transcriptome data series, representing over 1.8 million individual profiles ^1^. Altogether publically available molecular and cellular phenotyping data of all types constitute the biomedical research community’s “collective data”. Collective data can and should be exploited not only by researchers who have acquired valuable background in quantitative sciences, but also by “mainstream” life scientists, whose research can also greatly benefit from this vast resource. A unique global perspective can for instance simply be gained from examining the abundance of a single analyte across tens or hundreds of “omics” studies. In this report potential gaps in knowledge pertaining to the role of the ADAM9 were investigated through interpretation of changes in abundance of ADAM9 RNA across public transcriptome datasets relevant to human immunology.

“ADAM metallopeptidase 9 (ADAM9) is a member of the ADAM (a disintegrin and metalloprotease domain) family. Members of this family are membrane-anchored proteins structurally related to snake venom disintegrins, and have been implicated in a variety of biological processes involving cell-cell and cell-matrix interactions, including fertilization, muscle development, and neurogenesis. The protein encoded by this gene interacts with SH3 domain-containing proteins, binds mitotic arrest deficient 2 beta protein, and is also involved in TPA-induced ectodomain shedding of membrane-anchored heparin-binding EGF-like growth factor. Several alternatively spliced transcript variants have been identified for this gene.” (Quoted from RefSeq ^2^).

**Supplementary Figure 1.**
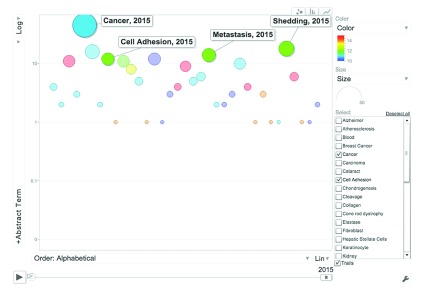
ADAM9 Literature Profile. This bubble graph indicates the frequency of abstracts of papers published on ADAM9 containing curated terms (Y-axis: Abstract Term) as of January 2015. Terms are ordered alphabetically along the X-axis. The colors indicate different categories (Blue: Cell type, Green: Disease, Red: Tissue, Light blue: Cellular component, Yellow: Function. The size of the bubbles is proportional to the number of abstract terms. **(Interactive version: Additional file iFIGURE 1 - X-axis drop down menu, select “Order: Alphabetical”; Y-axis drop down menu, select Log Scale; Click “Play” (bottom left corner))**.

**ADAM9 top functions include cellular adhesion, protein cleavage and shedding.** ( Supplementary Figure 1). Human ADAM9 protein cleaves and releases collagen XVII from the surface of skin keratinocytes ^3^. This activity is enhanced in the presence of reactive oxygen species. Mouse ADAM9 protein cleaves and releases epidermal growth factor (EGF) and fibroblast growth factor receptor 2IIIb (FGFR2IIIb) from the surface of prostate epithelial cells ^4^. Following LPS treatment, ADAM9 protein catalytic domain cleaves Angiotensin-I converting enzyme (ACE) from the surface of endothelial cells ^5^. Human ADAM9 protein disintegrin-cysteine-rich domain binds integrins and thus mediates cell adhesion ^6^. Human ADAM9 protein enhances adhesion and invasion of non-small lung tumors which mediates tumor metastasis ^7^. Mouse ADAM9 protein enhances tissue plasminogen activator (TPA)-mediated cleavage of CUB domain-containing protein 1 (CDCP1) ^8^. This activity mediates lung tumor metastasis. Human ADAM9 protein mediates cell-cell contact interaction between stromal fibroblasts and melanoma cells at the tumor-stroma border, thus contributing to proteolytic activities required during invasion of melanoma cells ^9^.

**ADAM9 expression and regulation.** ADAM9 has been reported as being expressed in various cell populations including monocytes ^10^, activated macrophages ^11^, epithelial cells, activated vascular smooth muscle cells, fibroblasts ^9^, keratinocytes and tumor cells. The abundance of ADAM9 RNA measured by RT-PCR is decreased *in vitro* in human melanoma cells after culture with collagen type I or with Interleukin 1 alpha (IL1α) compared to mock stimulated conditions ^12^.

**ADAM9 has been involved in disease processes including cancer, cone rod dystrophy and atherosclerosis.** Homozygous mutation of the human ADAM9 gene results in severe cone rod dystrophy and cataract ^13^. Mutation of the mouse ADAM9 gene results in no major abnormalities during development and adult life ^14^. The abundance of ADAM9 RNA and protein measured by immunostaining and RT-PCR is increased *in vivo* in human prostate tumors compared to normal tissue ^15^. The abundance of ADAM9 RNA measured by microarray and RT-PCR is increased *in vivo* in human advanced atherosclerotic plaque macrophages compared to normal tissue ^16^. This increase is predictive of Prostate Specific Antigen (PSA) relapse.

It is known that ADAM9 is upregulated in some tumor cells during pathologic processes and also contributes to the formation of multinucleate giant cells from monocytes and macrophages ^11^. However, little is known about the activities of ADAM9 in regulating physiologic or pathologic processes, especially during acute infection or in response to tissue damage.

## Methods

### ADAM9 bibliography screening and literature profiling

Existing knowledge pertaining to ADAM9 was retrieved using NCBI’s National Library of Medicine’s Pubmed search engine with a query that included official gene symbol and name as well as aliases: “ADAM9 OR ADAM-9 OR "ADAM metallopeptidase domain 9" OR MCMP OR MDC9 OR CORD9”. As of January of 2015, 287 papers were returned when running this query. By reviewing this literature keywords were identified that were classified under six categories corresponding to cell types, diseases, functions, tissues, molecules or processes. Frequencies of these keywords were then determined for the ADAM9 bibliography as shown in Supplementary Figure 1. This literature screen identified and prioritized existing knowledge about the gene ADAM9 and was used to prepare the background section of this manuscript and provided the necessary perspective for the interpretation of ADAM9 profiles across other large-scale datasets.

### Interactive data browsing application

We employed a resource that is described in details ^17^ and is available publicly: https://gxb.benaroyaresearch.org/dm3/landing.gsp. Briefly: we have assembled and curated a collection of 172 datasets that are relevant to human immunology, representing a total of 12,886 unique transcriptome profiles. These sets were selected among studies currently available in NCBI’s Gene Expression Omnibus (GEO, http://www.ncbi.nlm.nih.gov/geo/).

The custom software interface provides the user with a means to easily navigate and filter the compendium of available datasets ( https://gxb.benaroyaresearch.org/dm3/geneBrowser/list) ^17, 18^. Datasets of interest can be quickly identified either by filtering on criteria from pre-defined lists on the left or by entering a query term in the search box at the top of the dataset navigation page, we also provided the GXB tutorial in YouTube video; https://www.youtube.com/playlist?list=PLtx3tvfIzJ9XkRKUz6ISEJpAhqKyuiCiD.

### Graphical legends

Diagrams have been incorporated within each figure. These have a dual purpose, first they provide readers with a graphical summary of the findings and second constitute an attempt a structuring information for future computational applications. Indeed, an important limitation of communicating biomedical knowledge in the form of research articles is that it consists in unstructured information (free text). This type of information is notoriously difficult to extract by computational means ^19^. Standardized graphical summaries such as the ones provided in this manuscript constitute structured information that is both human readable and computationally tractable. The need for solutions will become more pressing as the biomedical literature continues to grow exponentially to such scales that it can only be very narrowly apprehended by research investigators. The graphical legends presented here merely serve as proof of concept.

### Statistical analyses

All statistical analyses were performed using GraphPad Prism software version 6 (GraphPad Software, San Diego, CA).

## Results and discussion

Raw data of ADAM9 transcripts in blood in response to tissue damage All primary data presented in this manuscript are provided as data files. Detailed legends for each data file can be found in the text file ‘Description of GSE datasets’. Click here for additional data file. Copyright: © 2016 Rinchai D et al. 2016 Data associated with the article are available under the terms of the Creative Commons Zero "No rights reserved" data waiver (CC0 1.0 Public domain dedication).

### Knowledge gap assessment

**Figure 1.**
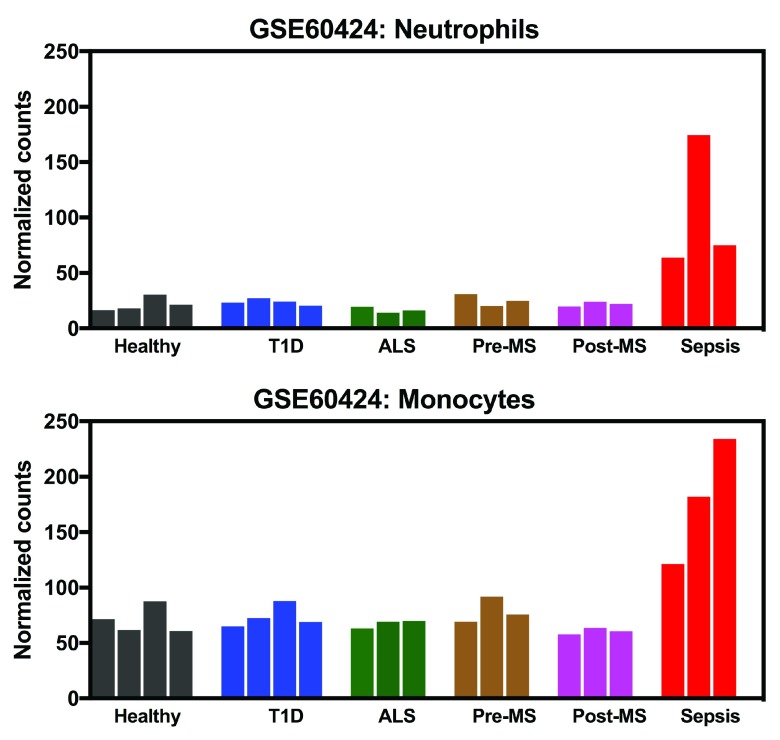
Elevated abundance of ADAM9 transcript in human monocytes and neutrophils during sepsis. The bar graphs show the abundance of ADAM9 transcripts measured by RNA-seq in neutrophils (top) and monocytes (bottom) isolated from blood collected in healthy donors (Black), patients with Type 1 diabetes (Blue), patients with ALS (Green), patients with multiple sclerosis pre- (Pre-MS; Brown) and post- (Post-MS; Pink) interferon treatment and patients with acute infections (sepsis; Red, caused by *Clostridium difficile, E. coli* and Meningococcal bacteria, respectively).

The seminal discovery was made while examining RNAseq transcriptional profiles. A knowledge gap was exposed when those results were interpreted in light of existing knowledge reported in the literature. Next, the initial observation was validated and further extended by examining profiles of the gene of interest, ADAM9, across a large number of independent publically available transcriptome datasets. The completion of these tasks was aided by a custom data browsing application loaded with a curated compendium of 172 datasets relevant to human immunology sourced from the National Center for Biotechnology Information’s (NCBI) Gene Expression Omnibus (GEO) ( https://gxb.benaroyaresearch.org/dm3/landing.gsp) ^17^. Briefly, ADAM9 transcript was identified as a potential early stage discovery while browsing RNA-sequencing profiles of blood leukocyte populations ( https://gxb.benaroyaresearch.org/dm3/geneBrowser/show/396), with the genes being ranked in alphabetical order. In this particular dataset whole blood sample of healthy donors, patients during acute infections (meningococcal sepsis, *E. coli* sepsis, *C. difficile* colitis), multiple sclerosis patients pre- and post- interferon treatment, patients with Type 1 diabetes and patients with amyotrophic lateral sclerosis (ALS) were obtained and monocyte, neutrophil, CD4 T cell, CD8 T cells, B cell, NK Cell isolated prior to profiling via RNA sequencing ^20^. The abundance of ADAM9 RNA measured by RNA-seq in human blood neutrophils and monocyte samples from subjects with sepsis was found to be markedly increased as compared to uninfected controls ( Figure 1; [ iFigure/ GSE60424] ^20^). By comparison levels of abundance of ADAM9 RNA in lymphocytes and Natural Killer (NK) cells were low and no changes were observed in subjects with sepsis in these cell populations. Despite the small number of septic subjects included in the study (N=3) the robust increase in abundance that was observed prompted attempts to validate and further extend this initial observation in independent public datasets that were part of the compendium.

### The abundance of ADAM9 increases during infection

Our data browsing tool allows the assessment of expression profiles across transcriptome datasets ( https://gxb.benaroyaresearch.org/dm3/geneBrowser/list). In order to validate and extend our original observation we looked up ADAM9 transcriptome profiles for all available 172 datasets ( https://gxb.benaroyaresearch.org/dm3/geneBrowser/crossProject?probeID=ENSG00000168615&geneSymbol=ADAM9&geneID=8754studies).

**Figure 2.**
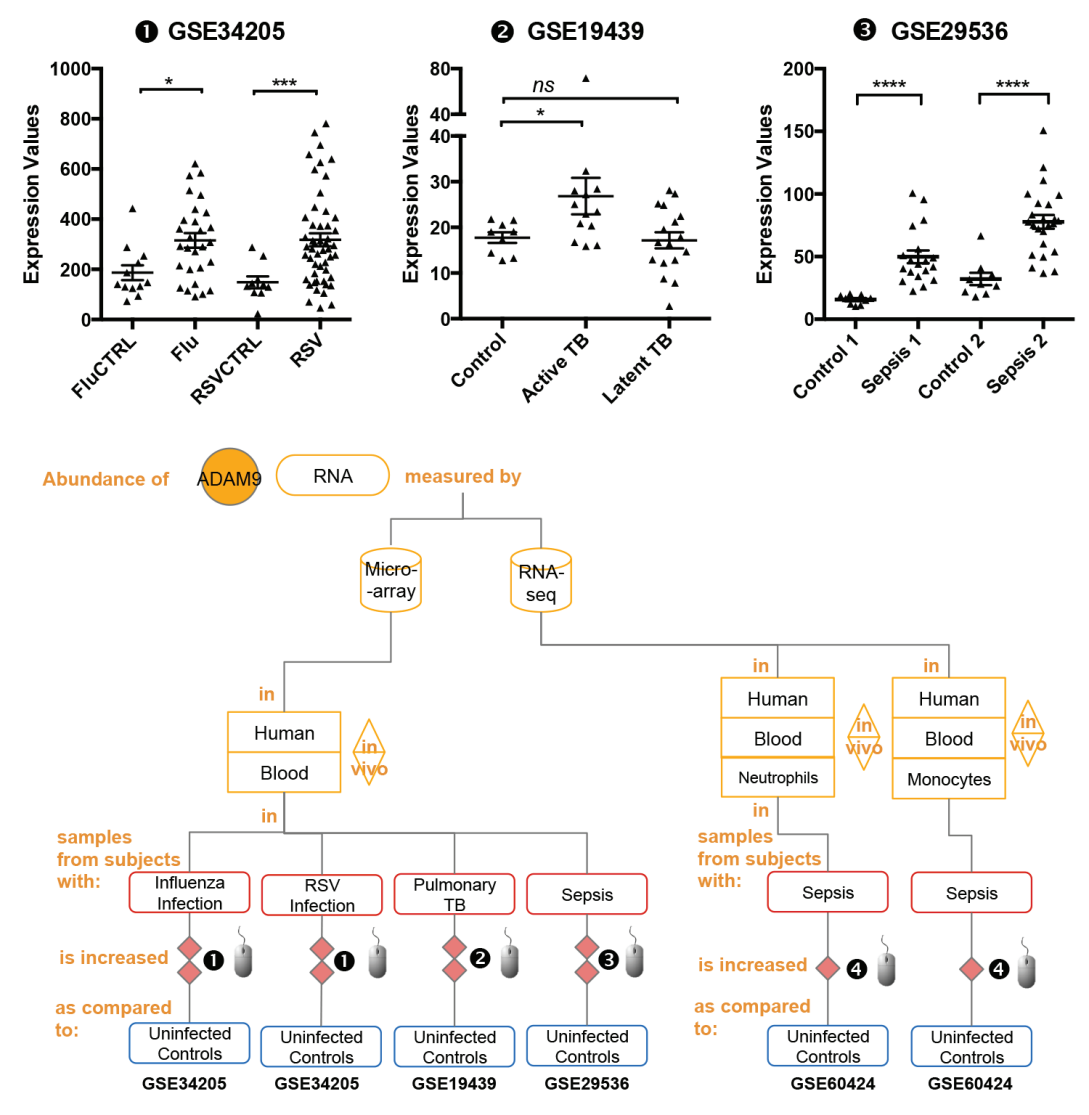
The abundance of ADAM9 increases during infection. mRNA expression levels for ADAM9 was measured by microarrays in whole blood obtained from children hospitalized with acute RSV and influenza virus infection (GSE34205), pulmonary tuberculosis patients (GSE19439) and patients with sepsis (GSE29536). The graphical legend represents visually the information associated with the public datasets used for the meta-interpretation of ADAM9 transcriptional profiles. The flow chart indicates how data were generated. Diamonds indicate availability of supporting data and in the interactive version are hyperlinked to context-rich interactive plots. Links to these plots are also provided below: ❶ **GSE34205:** In this study gene expression profiles were obtained from the whole blood of critically ill pediatric patients ^23^, Children hospitalized with acute RSV and influenza virus infection were offered study enrollment after microbiologic confirmation of the diagnosis. Blood samples were collected within 42–72 hours of hospitalization. Median age of subjects was 2.4 months (range 1.5–8.6). Uninfected subjects of similar demographics were recruited in the study and served as age-matched controls. Children with suspected or proven polymicrobial infections, with underlying chronic medical conditions (i.e congenital heart disease, renal insufficiency), with immunodeficiency, or those who received systemic steroids or other immunomodulatory therapies were excluded. As stated in the manuscript: “The Institutional Review Boards at the University of Texas Southwestern Medical Center and Baylor Institute for Immunology Research approved this study, and informed consent was obtained from legal guardians prior to any study-related procedure.” More details are available via the interactive data browsing application under the “study” tab. https://gxb.benaroyaresearch.org/dm3/miniURL/view/Ka ❷ **GSE19439:** Whole blood was collected from patients with different spectra of tuberculosis (TB) disease and healthy controls ^25^. All patients were sampled prior to the initiation of any anti-mycobacterial therapy. Active Pulmonary TB: all patients confirmed by isolation of *Mycobacterium tuberculosis* on culture of sputum or bronchoalvelolar lavage fluid. Latent TB: All patients were positive by tuberculin skin test (>14mm if BCG vaccinated, >5mm if not vaccinated) and were also positive by Interferon-Gamma Release assay (IGRA). As stated in the manuscript: “The local Research Ethics Committees (REC) at St Mary’s Hospital, London, UK approved the study”. https://gxb.benaroyaresearch.org/dm3/miniURL/view/Kb ❸ **GSE29536:** Whole blood was collected from culture positive patients meeting criteria for sepsis enrolled in two independent cohorts (Sepsis 1 and Sepsis 2) ^22^. Uninfected controls recruited in this study were of similar demographics. As stated in the manuscript: “The study was performed by recruitment of patients who were suspected of having hospital or community acquired infection. Clinical specimens were collected for bacterial culture within 24 hours following the diagnosis of sepsis. All blood samples were obtained at the Khon Kaen Regional Hospital, Khon Kaen, Thailand as approved by Khon Kaen University Ethic Committee for Human Research (Project number HE470506)”. https://gxb.benaroyaresearch.org/dm3/miniURL/view/Jl ❹ **GSE60424:** Whole blood sample of healthy donors, patients during acute infections (meningococcal sepsis, *E. coli* sepsis, *C. difficile* colitis), multiple sclerosis patients pre- and 24 hours post- interferon treatment, patients with Type 1 diabetes and patients with ALS were obtained and monocyte, neutrophil, CD4 T cell, CD8 T cells, B cell, NK Cell isolated prior to profiling via RNA sequencing ^21^. https://gxb.benaroyaresearch.org/dm3/miniURL/view/Kc Statistical significance was determined using Mann-Whitney U test. *ns, not significant*, * p < 0.05, *** p < 0.001 and *** *p < 0.0001*. The horizontal lines indicate mean ± standard errors (SE).

**Supplementary Figure 2.**
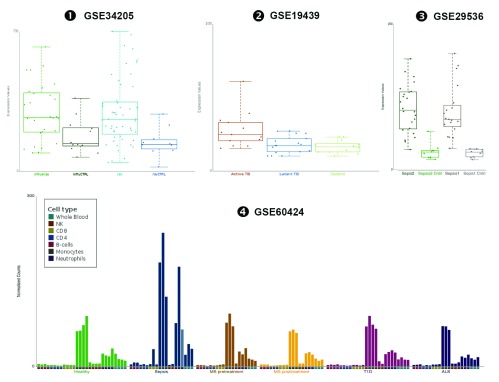
These plots are exported directly from the GXB tool and show data supporting the notion that the abundance of ADAM9 increases during infection as presented in Figure 2 of the manuscript. The interactive version of this plot can be accessed via the links provided below:
❶
https://gxb.benaroyaresearch.org/dm3/miniURL/view/Ka
❷
https://gxb.benaroyaresearch.org/dm3/miniURL/view/Kb
❸
https://gxb.benaroyaresearch.org/dm3/miniURL/view/Jl
❹
https://gxb.benaroyaresearch.org/dm3/miniURL/view/Kc

The abundance of ADAM9 RNA measured by microarrays in human blood samples was significantly increased as compared to uninfected controls in subjects with sepsis [ iFigure/ GSE28750] ^21^ & [ iFigure/ GSE29536] ^22^, in subjects with bacterial and influenza pneumonia [ iFigure/ GSE34205] ^23^, [ iFigure/ GSE40012] ^24^, in subjects with respiratory syncytial virus (RSV) infection [ iFigure/ GSE34205] ^23^ & [ iFigure/ GSE17156] ^23^ and in subjects with tuberculosis [ iFigure/ GSE19439] ^25^ & [ iFigure/ GSE34608] ^26^. Aggregated findings were reported in the form of flow charts that were generated using google docs presentations, with links to the source interactive graphs systematically provided as hyperlinks ( Figure 2, Supplementary Figure 2 and Table 1). Altogether these data indicate that increase in abundance of ADAM9 can be detected in blood leukocytes, including monocytes and neutrophils fractions during bacterial and viral infection.

**Table 1.** Increased abundance of ADAM9 during infection.

GEO ID	A vs B	Avg A-Avg B	Avg A/Avg B	P value
GSE34205	Influenza vs Influenza CTRL	129.0	1.7	*0.0144*
	RSV vs RSV CTRL	169.4	2.1	*0.0009*
GSE19439	Active TB vs Control	9.1	1.5	*0.0169*
	Latent TB vs Control	-0.6	1.0	0.8688
GSE29536	Sepsis 1 vs Control	34.1	3.2	*< 0.0001*
	Sepsis 2 vs Control	45.6	2.4	*< 0.0001*
GSE60424	Sepsis vs Control (Neutrophil)	82.7	4.6	*0.0380*
	Sepsis vs Control (Monocyte)	108.6	2.5	*0.0121*

**Note :** Avg = average abundance of ADAM9 within a given group. Statistical significance was determined using Mann-Whitney U test.

### The abundance of ADAM9 increases only marginally following treatment with pathogen-associated molecules

**Figure 3.**
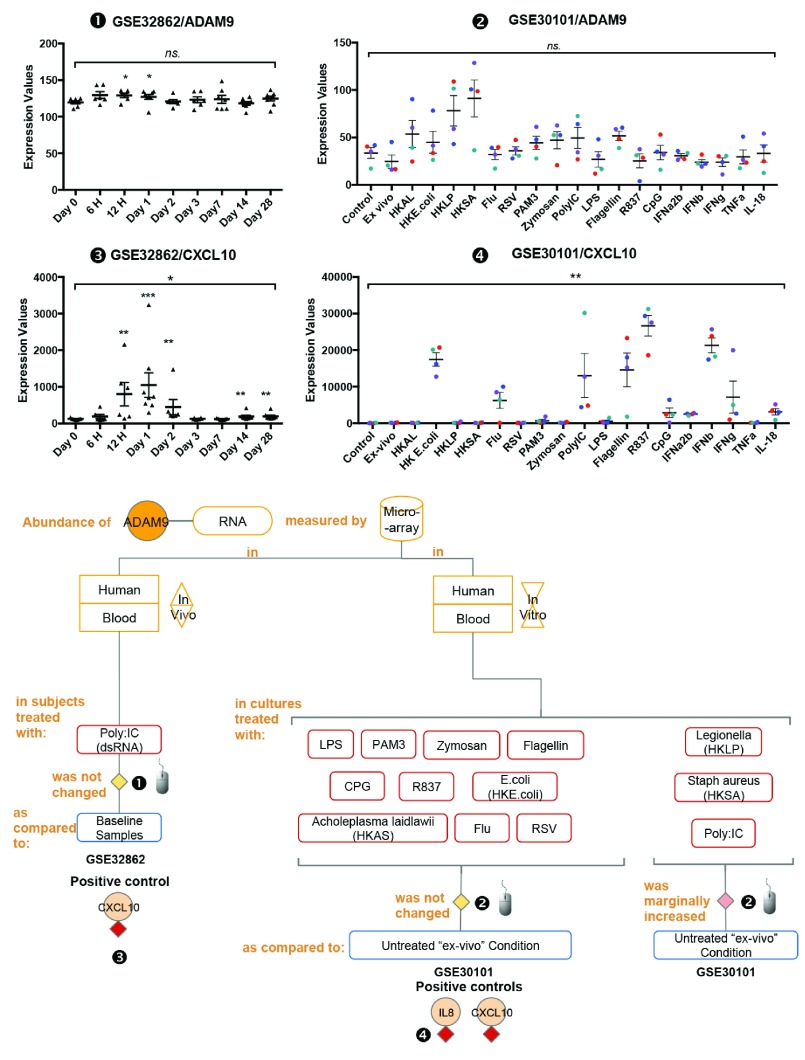
The abundance of ADAM9 increases only marginally following treatment with pathogen-associated molecules. mRNA expression levels for ADAM9 and CXCL10 were measured by microarrays in whole blood obtained from 8 healthy volunteers following sub-cutaneous administration of synthetic dsRNA (poly:IC) or placebo at baseline (day 0) and at 6 and 12 hours and 1, 2, 3, 7, 14, and 28 days (GSE32862), and from 4 healthy individuals(shown indifferent colors; Red, blue, green and purple) and stimulated *in vitro* for 6 hours with a wide range of immune stimuli including PAM3, Zymosan, Poly IC, E-LPS, Flagellin, R837, CpG Type A, heat-killed *Legionella pneumophila* (HKLP), heat-killed *Acholeplasma laidlawii* (HKAL), and heat-killed *Staphylococcus aureus* (HKSA); IL-18, TNF-α, IFN-α2b, IFN-β, IFN-γ; heat-killed *Escherichia coli* (HKEC), live influenza A virus and live (GSE30101). The graphical legend represents visually the information associated with the public datasets used for the meta-interpretation of ADAM9 transcriptional profiles. The flow chart indicates how data were generated. Diamonds indicate availability of supporting data and in the interactive version are hyperlinked to context-rich interactive plots. Links to these plots are also provided below: ❶ **GSE32862** Whole blood was collected from 8 healthy volunteers following sub-cutaneous administration of synthetic dsRNA (poly:IC) or placebo at baseline (day 0) and at 6 and 12 hours and 1, 2, 3, 7, 14, and 28 days. As stated in the manuscript: “The study was approved by the Institutional Review Board of The Rockefeller University Hospital. Individual participants in this study provided written informed consent after appropriate review, discussion, and counseling by the clinical study team” ^28^. https://gxb.benaroyaresearch.org/dm3/miniURL/view/Kd ❷ **GSE30101** Blood was collected from four healthy individuals and stimulated *in vitro* for 6 hours with a wide range of immune stimuli including PAM3, Zymosan, Poly IC, E-LPS, Flagellin, R837, CpG Type A, HKLP, HKAL, and HKSA; IL-18, TNF-α, IFN-α2b, IFN-β, IFN-γ; HKEC, live influenza A virus and live RSV. As stated in the manuscript: “The study was approved by the Baylor Research Institute Institutional Review Board at Baylor University Medical Center (Dallas, TX)” ^27^. http://www.interactivefigures.com:80/dm3/miniURL/view/KB ❸ See description above. https://gxb.benaroyaresearch.org/dm3/miniURL/view/Jr ❹ See description above. http://www.interactivefigures.com:80/dm3/miniURL/view/Jw Statistical significance was determined using one-way ANOVA and Dunnett’s multiple comparisons test. *ns, not significant*, * p < 0.05, ** p < 0.01, and *** *p < 0.001*. The horizontal lines indicate mean ± standard errors (SE).

**Supplementary Figure 3.**
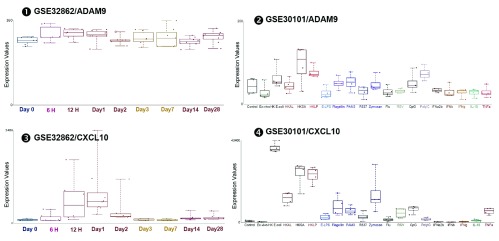
These plots are exported directly from the GXB tool and show data supporting the notion that the abundance of ADAM9 increases only marginally following treatment with pathogen-associated molecules as presented in Figure 3 of the manuscript. The interactive version of this plot can be accessed via the links provided below:
❶
https://gxb.benaroyaresearch.org/dm3/miniURL/view/Kd
❷
http://www.interactivefigures.com:80/dm3/miniURL/view/KB
❸
https://gxb.benaroyaresearch.org/dm3/miniURL/view/Jr
❹
http://www.interactivefigures.com:80/dm3/miniURL/view/Jw

Next, we investigated the regulation of ADAM9 transcription following leukocyte exposure to pathogens and pathogen-associated molecules. The abundance of ADAM9 RNA measured by microarrays in human blood cultures treated with Heat Killed *E.coli*, Heat Killed *Staphylococcus aureus* (HKSA) or Heat Killed *Legionella pneumophillum* (HKLP) for 6 hours was increased marginally as compared to unstimulated conditions [ iFigure/ GSE30101] ^27^. The abundance of ADAM9 RNA measured by microarrays in human blood cultures treated with Heat Killed *Acholeplasma laidlawii* (HKAS), *E. coli* LPS (E-LPS), Flagellin, PAM3, R837, Zymosan, Influenza virus, RSV, CpG, Poly:IC, for 6 hours was not changed as compared to unstimulated conditions ( *Ex-vivo*) [ iFigure/ GSE30101] ^27^; IL8 [ iFigure] and CXCL10 [ iFigure] serve as positive controls. The abundance of ADAM9 RNA measured by microarrays in human blood samples from subjects treated with poly:IC for 1 day was marginally increased as compared to baseline samples [ iFigure/ GSE32862] ^28^; CXCL10 [ iFigure] serves as a positive control ( Figure 3 and Supplementary Figure 3). Statistical analysis results are shown in Table 2. Taken together, these results showed that the abundance of ADAM9 was not changed or changed only marginally after stimulation with purified molecules bearing Pathogen Associated Molecular Patterns (PAMPs). These finding raised the question as to whether ADAM9 transcription might be activated instead by host-derived Damage-Associated Molecular Pattern molecule (DAMPs) ^29, 30^.

**Table 2.** Increased abundance of ADAM9 following treatment with PAMPs.

GEO ID	A vs B	Avg A-Avg B	Avg A/Avg B	P value
GSE32682	Day 0 VS 6 H	10.0	1.1	*0.0734*
(ADAM9)	Day 0 VS 12 H	9.5	1.1	*0.0350*
	Day 0 VS Day 1	7.5	1.1	*0.0140*
	Day 0 VS Day 2	1.1	1.0	*0.9172*
	Day 0 VS Day 3	3.7	1.0	*0.7133*
	Day 0 VS Day 7	4.3	1.0	*0.6894*
	Day 0 VS Day 14	-1.2	1.0	*0.9305*
	Day 0 VS Day 28	5.3	1.0	*0.1504*
GSE32682	Day 0 VS 6 H	66.9	1.5	*0.4727*
(CXCL10)	Day 0 VS 12 H	676.3	6.5	*> 0.9999*
	Day 0 VS Day 1	924.2	8.5	*0.0023*
	Day 0 VS Day 2	324.0	3.6	*0.0003*
	Day 0 VS Day 3	5.5	1.0	*0.7133*
	Day 0 VS Day 7	-3.8	1.0	*0.8718*
	Day 0 VS Day 14	67.5	1.5	*0.0093*
	Day 0 VS Day 28	71.0	1.6	*< 0.0059*

**Note :** Avg = average abundance of ADAM9 within a given group. Statistical significance was determined using Mann-Whitney U test.

### The abundance of ADAM9 increases during tissue remodeling

**Figure 4.**
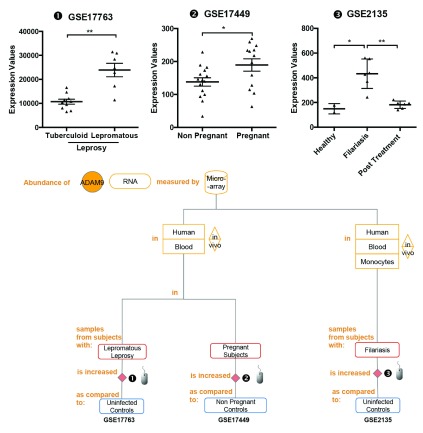
The abundance of ADAM9 increases during tissue remodeling. mRNA expression levels for ADAM9 was measured by microarrays in skin biopsies were obtained from patients with leprosy classified as tuberculoid leprosy or lepromatous leprosy (GSE17763), Peripheral Blood Mononuclear Cells obtained from 12 women (7 MS patients and 5 healthy controls) followed during their pregnancy (GSE17449) and monocyte from flilariasis before and after treatment (GSE2135). The graphical legend represents visually the information associated with the public datasets used for the meta-interpretation of ADAM9 transcriptional profiles. The flow chart indicates how data were generated. Diamonds indicate availability of supporting data and in the interactive version are hyperlinked to context-rich interactive plots. Links to these plots are also provided below: ❶ **GSE17763** Skin biopsies were obtained from patients with leprosy classified as tuberculoid leprosy (controlled disease, few skin lesions) or lepromatous leprosy (uncontrolled diseases, widespread lesions) ^31^. All tuberculoid and lepromatous specimens were taken at the time of diagnosis before treatment, and reversal reaction biopsies (labeled as “reaction”) were taken upon follow from patients originally diagnosed with borderline lepromatous leprosy (UCLA Institutional Review Board # 92-10-591-31). https://gxb.benaroyaresearch.org/dm3/miniURL/view/Ke ❷ **GSE17449** Peripheral Blood Mononuclear Cells were isolated from the blood of 12 women (7 MS patients and 5 healthy controls) followed during their pregnancy. As stated in the manuscript: This study was approved by the Ethical Committee of the San Luigi University Hospital (March 2006, approval n°87)” ^32^. Samples were obtained before pregnancy and at 9 months. https://gxb.benaroyaresearch.org/dm3/miniURL/view/KD ❸ **GSE2135** Monocytes were isolated from the peripheral blood of patently infected filaria patients (either *Wuchereria bancrofti*, *Mansonella perstans*, or both), and from uninfected blood bank donors in Mali. As stated in the manuscript: “All study participants signed informed consent forms, and all protocols were approved by the institutional review boards at both the NIAID and the University of Mali (Bamako)” ^33^. Samples were collected from infected patients prior to and after antifilarial treatment. https://gxb.benaroyaresearch.org/dm3/miniURL/view/KB Statistical significance was determined using Mann-Whitney U test. *ns, not significant*, * *p < 0.05* and *** p < 0.001. The horizontal lines indicate mean ± standard errors (SE).

**Supplementary Figure 4.**
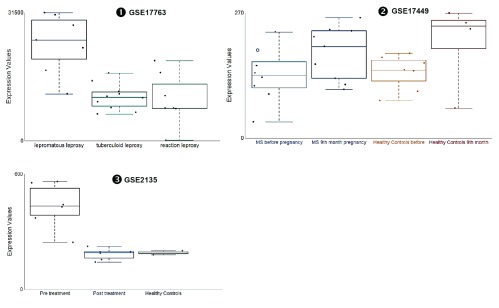
These plots are exported directly from the GXB tool and show data supporting the notion that the abundance of ADAM9 increases during tissue remodeling as presented in Figure 4 of the manuscript. The interactive version of this plot can be accessed via the links provided below:
❶
https://gxb.benaroyaresearch.org/dm3/miniURL/view/Ke
❷
https://gxb.benaroyaresearch.org/dm3/miniURL/view/KD
❸
https://gxb.benaroyaresearch.org/dm3/miniURL/view/KB

Our dataset screen revealed in addition that changes in abundance of ADAM9 could be associated with tissue remodeling. The abundance of ADAM9 RNA measured by microarrays in human skin biopsy samples of subjects with lepromatous leprosy was significantly increased as compared to controls in subjects with tuberculoid leprosy [ iFigure/ GSE17763] ^31^. The abundance of ADAM9 RNA measured by microarrays in human blood samples was significantly increased as compared to controls in pregnant subjects [ iFigure/ GSE17449] ^32^. The abundance of ADAM9 RNA measured by microarrays in human blood monocytes samples from subjects with filariasis was significantly increased as compared to uninfected controls [ iFigure/ GSE2135] ^33^. These results are shown in Table 3, Figure 4 and Supplementary Figure 4. A common thread between these different states is that they involve extensive tissue remodeling, whether it involves the skin (leprosy), placental tissue (pregnancy) or lymphatic tissues (filariasis).

**Table 3.** Increased abundance of ADAM9 during tissue remodeling.

GEO ID	A vs B	Avg A-Avg B	Avg A/Avg B	P value
GSE17763	Lepromatous leprosy VS Tuberculoid leprosy	13164.0	2.2	*0.0012*
GSE17449	Non pregnancy VS Pregnancy	51.3	1.4	*0.0366*
GSE2135	Filariasis VS Post Treatment	251.1	2.4	*0.0313* ^*^
	Filariasis VS Healthy Control	283.6	2.9	*0.0197*

**Note :** Avg = average abundance of ADAM9 within a given group. Statistical significance were determined using Mann-Whitney U test. * (Pair samples) Statistical significance was determined using Wilcoxon test.

### The abundance of ADAM9 increases following tissue injury and sterile inflammation

**Figure 5.**
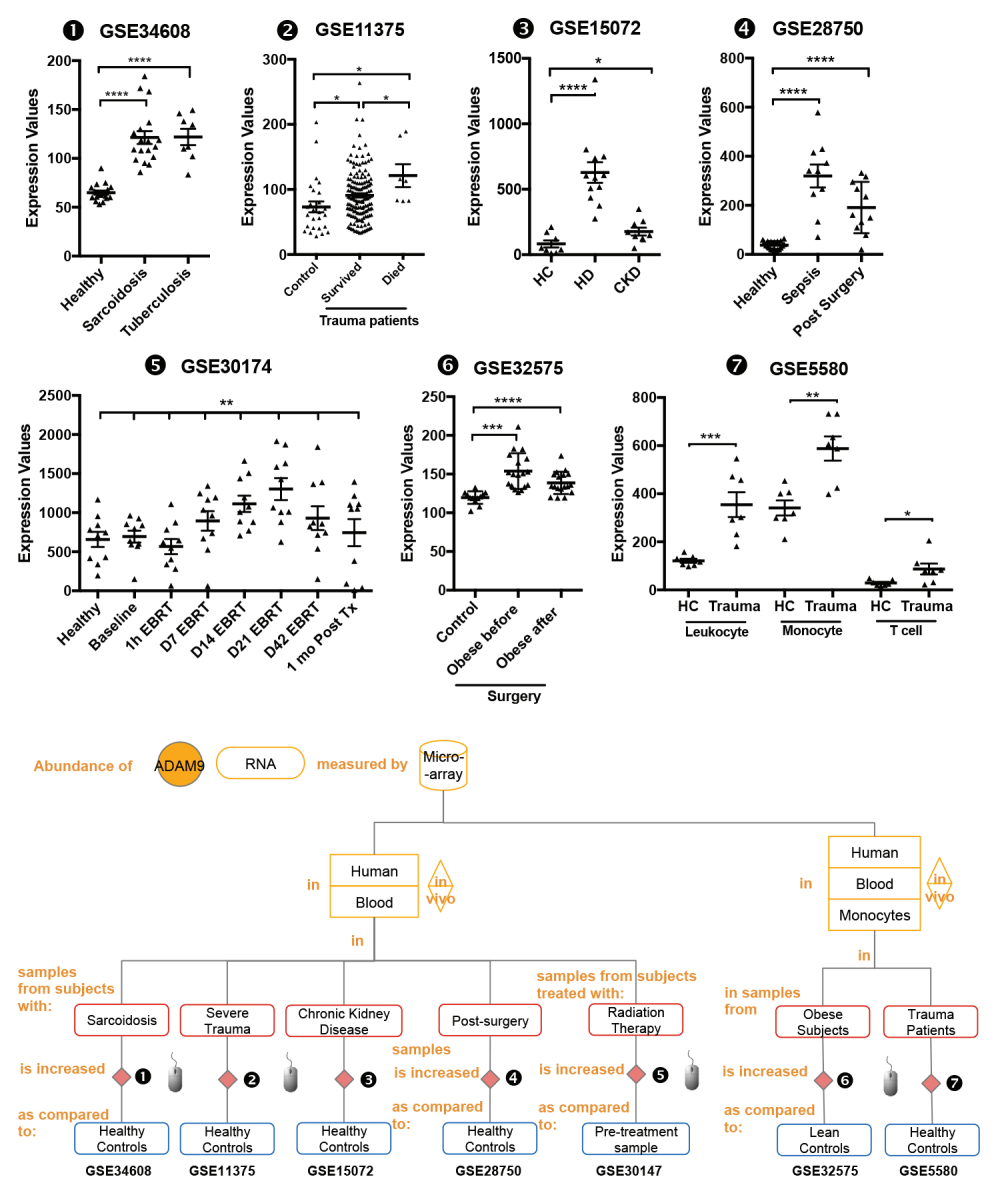
The abundance of ADAM9 increases following tissue injury and sterile inflammation. mRNA expression levels for ADAM9 was measured by microarrays in whole blood obtained from patients with active tuberculosis and sarcoidosis (GSE34608), from patients following severe blunt trauma within 12 hours of traumatic injury who survive and non-survive (GSE11375), from blood of patients with stage II-III Chronic kidney disease (CKD), patients undergoing hemodialysis treatment (HD) and healthy controls (GSE15072), from septicemic patients (GSE28750), from 10 men with non-metastatic prostate cancer; baseline (before External Beam Radiation Therapy - EBRT); days 1, 7, 14, 21, 42 of EBRT; and 30 days post-EBRT (GSE30147) and monocyte from blood of 18 morbidly obese subjects before and three months after bariatric surgery (GSE32575) and from seven subjects with defined multi organ dysfunction syndrome that developed after experiencing severe traumatic injury (GSE5580). The graphical legend represents visually the information associated with the public datasets used for the meta-interpretation of ADAM9 transcriptional profiles. ( https://docs.google.com/presentation/d/12ytv11_LmMOAsocziIAe8MwwKOrGgHSO60hpdK2hHsQ/edit#slide=id.g496fd210c_046). The flow chart indicates how data were generated. Diamonds indicate availability of supporting data and in the interactive version are hyperlinked to context-rich interactive plots. Links to these plots are also provided below: ❶ **GSE34608** blood was collected from patients with active tuberculosis and sarcoidosis as well as uninfected controls. As stated in the manuscript: “The study was approved by the Ethical Committee 1 of the Charité University Medicine, Campus Mitte in Berlin, the University of Luebeck, and the University of Freiburg in Germany” ^26^. https://gxb.benaroyaresearch.org/dm3/miniURL/view/Jt ❷ **GSE11375** blood was collected from patients following severe blunt trauma within 12 hours of traumatic injury. As stated in the manuscript: “The study was approved by review board the Harborview Medical Center and the University of Texas Medical Branch-Galveston” ^34^. https://gxb.benaroyaresearch.org/dm3/miniURL/view/K8 ❸ **GSE15072** Peripheral Blood Mononuclear Cells were isolated from the blood of patients with stage II-III Chronic kidney disease (CKD), patients undergoing hemodialysis treatment (HD) and healthy controls. As stated in the manuscript, “The study was carried out according to the Declaration of Helsinki and approved by the institutional ethical board of the University Hospital ‘Policlinico di Bari’, Bari, Italy” ^35^. https://gxb.benaroyaresearch.org/dm3/miniURL/view/KE ❹ **GSE28750** Blood was collected from sepsis patients with clinical evidence of systemic infection based on microbiology diagnoses (n=10). Participants in the post-surgical (PS) group were recruited pre-operatively and blood samples collected within 24 hours following surgery (n=11). Healthy controls (HC) included hospital staff with no known concurrent illnesses (n=20). As stated in the manuscript: “The study protocol was approved by institutional review boards (IRBs)/Human Research Ethics Committees (HRECs) from Mater Health Services (MHS), Uniting Care, the Royal Brisbane & Women's Hospital and the Nepean Hospital Human Research Ethics Committee, prior to the recruitment of study volunteers” ^21^. https://gxb.benaroyaresearch.org/dm3/miniURL/view/K6 ❺ **GSE30174** Blood samples were collected from ten subjects at 7 timepoints for microarray analysis: baseline (before External Beam Radiation Therapy - EBRT); days 1, 7, 14, 21, 42 of EBRT; and 30 days post-EBRT. Baseline data obtained from subjects were compared to data obtained from age-, race-, and gender-matched healthy controls. As stated in the manuscript: “The protocols were approved by the Institutional Review Board of the National Institute of Health (NIH), Bethesda, Maryland, USA” ^36^. https://gxb.benaroyaresearch.org/dm3/miniURL/view/K4 ❻ **GSE32575** CD14+ monocytes were isolated from the blood of 18 morbidly obese subjects (BMI: 45.1±1.4 kg/m2) before and three months after bariatric surgery. Six lean age-matched female (BMI: 20.3±0.5 kg/m2, mean±SEM) were used as controls. As stated in the manuscript: “This study complies with the Declaration of Helsinki and the Medical Ethics Committee of the Katholieke Universiteit Leuven approved the study protocol. All human participants gave written informed consent” ^37^. https://gxb.benaroyaresearch.org/dm3/miniURL/view/K5 ❼ **GSE5580** Monocytes were isolated from the peripheral venous blood of seven subjects with defined multi organ dysfunction syndrome that developed after experiencing severe traumatic injury. Blood was also obtained from seven age-, sex-, and ethnicity-matched healthy subjects. As stated in the manuscript: “ Informed consent was obtained from 18 severely injured patients and 22 healthy subjects under a protocol approved by the University of Rochester School of Medicine Institutional Review Board” ^38^. https://gxb.benaroyaresearch.org/dm3/miniURL/view/KC Statistical significance was determined using Mann-Whitney U test or one-way ANOVA and Dunnett’s multiple comparisons test (GSE30174). *ns, not significant*, * p < 0.05, ** p < 0.01, and *** p < 0.001. The horizontal lines indicate mean ± standard errors (SE).

**Supplementary Figure 5.**
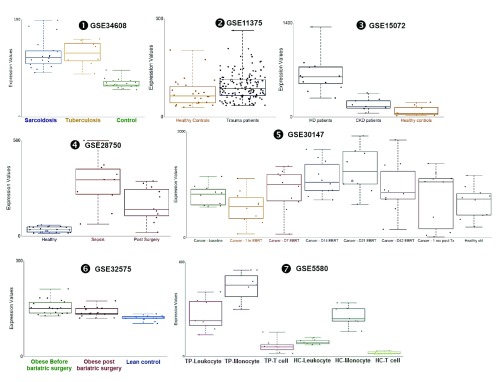
These plots are exported directly from the GXB tool and show data supporting the notion that the abundance of ADAM9 increases following tissue injury and sterile inflammation. The interactive version of this plot can be accessed via the links provided below:
❶
https://gxb.benaroyaresearch.org/dm3/miniURL/view/Jt
❷
https://gxb.benaroyaresearch.org/dm3/miniURL/view/K8
❸
https://gxb.benaroyaresearch.org/dm3/miniURL/view/KE
❹
https://gxb.benaroyaresearch.org/dm3/miniURL/view/K6
❺
https://gxb.benaroyaresearch.org/dm3/miniURL/view/K4
❻
https://gxb.benaroyaresearch.org/dm3/miniURL/view/K5
❼
https://gxb.benaroyaresearch.org/dm3/miniURL/view/KC

**Supplementary Figure 6.**
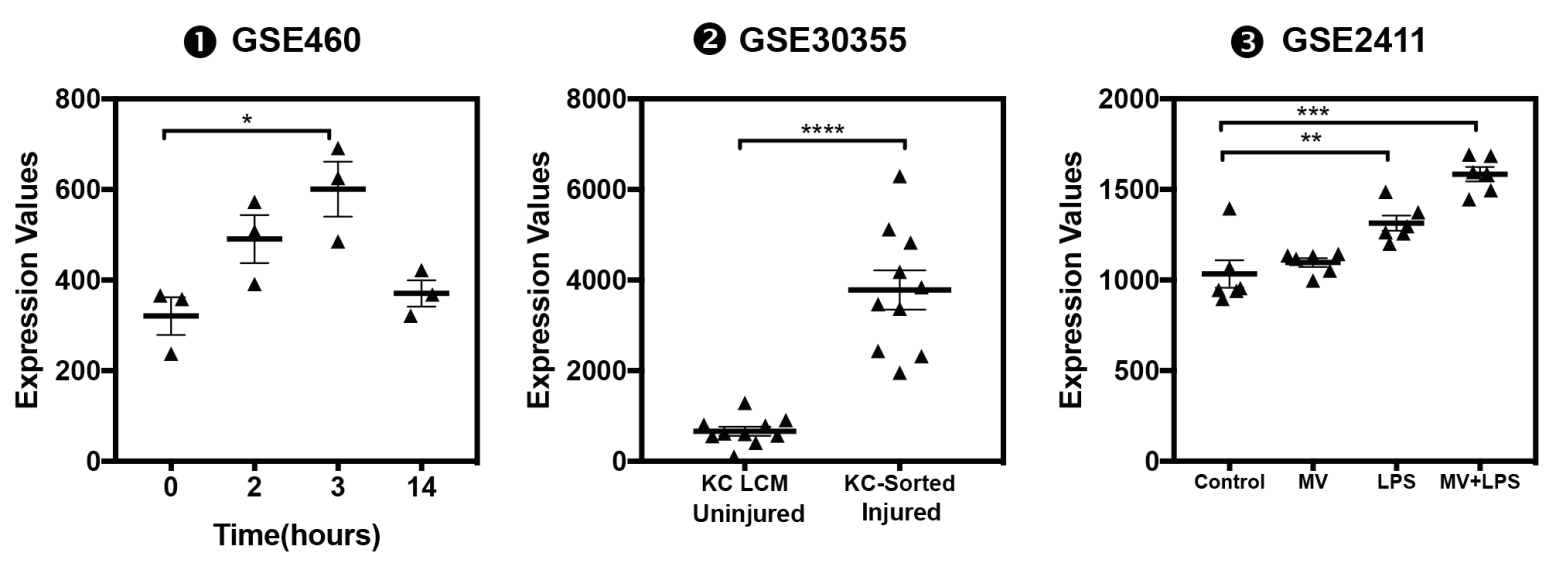
The abundance of ADAM9 increases in different models of tissue injury. mRNA expression levels for ADAM9 was measured by microarrays in a murine inflammatory lung injury and mechanical ventilation model (GSE2411), an *in vitro* human epidermal injury model ( GSE30355) and mouse thermal injury model ( GSE460). These raw data were exported from GEO and were plotted by GraphPad Prism. ❶ **GSE460** Thermal injury was induced in mice, and skin excised at 0 hours, 2 hours, 3 days and 14 days post-injury. https://www.ncbi.nlm.nih.gov/geo/tools/profileGraph.cgi?ID=GDS353:96738_at ❷ **GSE30355** Keratinocyte response to injury was evaluated in (1) epidermal samples isolated by laser capture microscopy and (2) cultured KCs https://www.ncbi.nlm.nih.gov/geo/tools/profileGraph.cgi?ID=GDS4608:1570042_a_at ❸ **GSE2411** Twenty four C57/B6 male mice were randomized to four groups: 1) Control, 2) mechanical ventilation (MV), 3) LPS, and 4) MV+LPS. Expression profiling of whole lungs revealed a significant augmentation of ADAM9 transcript levels in the combined MV+LPS group relative to the other 3 conditions. https://www.ncbi.nlm.nih.gov/geo/tools/profileGraph.cgi?ID=GDS1276:1416094_at

Changes in ADAM9 transcript abundance were observed in additional datasets: The abundance of ADAM9 RNA measured by microarrays in human blood samples was significantly increased as compared to healthy controls in subjects with sarcoidosis [ iFigure/ GSE34608] ^26^, in subjects after severe blunt trauma [ iFigure/ GSE11375] ^34^, in subjects with chronic kidney disease [ iFigure/ GSE15072] ^35^, and in subjects who have undergone elective thoracic or abdominal surgery [ iFigure/ GSE28750] ^21^. Furthermore, we found that the abundance of ADAM9 in trauma patients who did not survive (mean± 2SD; 121.3± 92.98) was significantly higher ( *p <0.05*) than those who survived (mean± 2SD; 90.86± 78.08) GSE11375. The abundance of ADAM9 RNA measured by microarrays in human blood samples from subjects treated with localized external beam radiation therapy for 42 days was significantly increased as compared to baseline samples [ iFigure/ GSE30174] ^36^. The abundance of ADAM9 RNA measured by microarrays in human blood monocytes samples from obese subjects was significantly increased as compared to lean controls [ iFigure/ GSE32575] ^37^. Finally, the abundance of ADAM9 RNA measured by microarrays in human blood monocytes samples from subjects after severe trauma was significantly increased as compared to healthy controls [ iFigure/ GSE5580] ^38^. These results showed that increase in ADAM9 transcript abundance was associated with tissue injury and sterile inflammation ( Table 4, Figure 5 and Supplementary Figure 5) and thus are consistent with the observations that are reported above associating increase in ADAM9 RNA with responses to Damage-Associated Molecular Pattern molecules (DAMPs) and tissue remodeling. Further evidence demonstrating the association of ADAM9 with tissue damage and injury was found in public transcriptome datasets generated by investigators employing mouse *in vivo* models and a human *in vitro* system ( supplementary Figure 6): 1) abundance of ADAM9 transcript increased over time at 0, 2 hours, 3 days following thermal injury in a murine dermal burn wound model ( GSE460); 2) An epidermal injury model ( GSE30355) showed that abundance of ADAM9 was significantly higher in injured epidermis (sorted human keratinocyte (KC)) in comparison to uninjured cells (laser capture microscopy or *in vitro* cultured keratinocytes) ^39^. And 3) abundance of ADAM9 transcripts was increased lungs of C57BL/6 mice that developed acute lung injury after exposure to low-dose LPS and mechanical ventilation ( GSE2411) in an vivo model of lung inflammation and injury ( GSE2411) ^40^.

**Table 4.** Increased abundance of ADAM9 following tissue injury and sterile inflammation.

GEO ID	A vs B	Avg A-Avg B	Avg A/Avg B	P value
GSE34608	Sarcoidosis VS Control	56.4	1.9	*< 0.0001*
	Tuberculosis VS control	56.9	1.9	*< 0.0001*
GSE11375	Survived VS Control	17.7	1.2	*0.0367*
	Died VS Control	45.2	1.6	*0.0226*
	Survived VS Died	27.5	1.3	*0.0473*
GSE15072	HD VS Healthy	545.6	7.6	*< 0.0001*
	CKD VS Healthy	94.3	2.1	*0.0359*
GSE28750	Post surgery VS Healthy	153.2	5.1	*< 0.0001*
	Sepsis VS Healthy	281.8	8.5	*< 0.0001*
GSE30174 **	Healthy VS Baseline	35.8	1.1	*0.7243*
	Healthy VS 1h EBRT	-91.3	0.9	*0.4727*
	Healthy VS D7 EBRT	236.5	1.4	*0.1419*
	Healthy VS D14 EBRT	455.8	1.7	*0.0068*
	Healthy VS D21 EBRT	643.8	2.0	*0.0021*
	Healthy VS D42 EBRT	272.1	1.4	*0.2150*
	Healthy VS 1 mo Post Tx	85.8	1.1	*0.5678*
GSE32575	Obese before surgery VS control	34.1	1.3	*< 0.0001*
	Obese post surgery VS control	19.0	1.2	*0.0001*
GSE5580	TP mono VS HC mono	247.1	1.7	*0.0070*
	TP Leukocyte VS HC Leukocyte	233.2	2.9	*0.0006*
	TP T cell VS HC T cell	57.9	3.0	*0.0175*

**Note :** Avg = average abundance of ADAM9 within a given group. Statistical significance was determined using Mann-Whitney U test. ** This dataset was tested by One-way ANOVA and Dunnett’s multiple comparisons test, P value summary = *0.0042*.

## Conclusions

**Figure 6.**
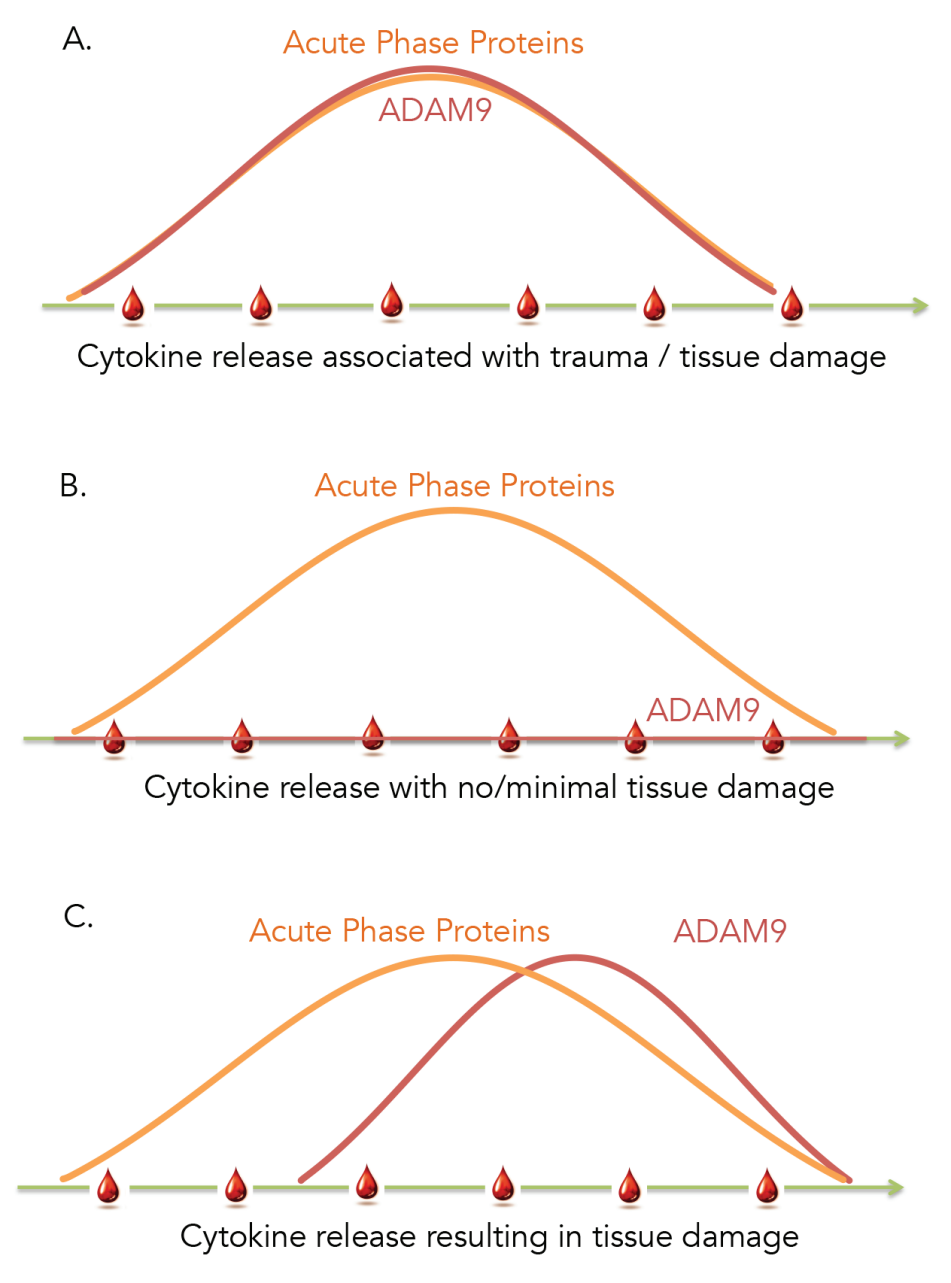
Proposed Model. **A**. Sterile inflammation resulting from tissue injury caused for instance by severe trauma, surgery or radiation therapy can be monitored via the use of prototypical markers of inflammation (acute phase proteins) with ADAM9 levels increasing in concert. **B**. Acute infection also causes a measurable inflammatory response that is the direct result of the antimicrobial response mounted by the immune system. This response can develop in absence of substantial tissue injury and thus does not cause an increase in abundance of ADAM9. **C**. When substantial tissue injury occurs as a result of the infection the abundance of ADAM9 rises, which detection enables the identification and triage of critically ill subjects.

This study is the first report describing the modulation of levels of ADAM9 transcripts in human whole blood and showing restriction of its expression to neutrophils and monocytes. In addition we observed that the abundance of ADAM9 was increased during acute infection but did not change after stimulation with pathogen-derived molecules. It was not changed *in vivo* following administration of synthetic double stranded RNA (polyIC), a treatment that mimics viral exposure. Notably, it was not increased either in patients during the early acute phase of HIV infection when an intense immunological response is detected in absence of clinical symptoms iFigure/ GSE29536] ^22^. However, ADAM9 transcript abundance was increased in the blood of patients as a result of tissue damage, sterile inflammation and tissue remodeling. Therefore, in addition to its widely reported role in the pathogenesis of cancer the constellation of findings that we are reporting point towards the involvement of ADAM9 in immune-mediated processes and suggest that ADAM9 may constitute a valuable marker for assessing tissue damage, whether it occurs as result of acute infection, traumatic injury or medical procedures such as surgery or radiation therapy. Furthermore, our observations may also be of high significance in the context of acute infections since unlike “generic” markers of inflammation, that could also be used to assess tissue injury in other settings, ADAM9 would not be confounded by the host responses to the pathogen and may therefore accurately reflect damage to the patient tissues or organs ( Figure 6). Thus ADAM9 blood transcript levels, or possibly levels of circulating proteins, could potentially be employed for triage of patients presenting with symptoms of infection in the emergency room or for monitoring of patients in intensive care units. The functional significance of elevated levels of this proteinase in blood of patients is unclear. While it has been associated with tissue repair increase in protein or transcript levels in the circulation may be an indication of catastrophic tissue damage that will lead to poor outcomes. This is suggested for instance by the fact that abundance of ADAM9 in patients who did not survive was significantly higher than those who survive (GSE11375 - profiling of responses in the blood of trauma patient). In another dataset GSE34205/GSE38900 (Viral infections) we also show that abundance of ADAM9 is correlated with degree of severity in pediatric viral infection (RSV, influenza and HRV infection), moreover level of ADAM9 transcript in patients who were ventilated were significantly higher than that who were non-ventilated.

Our analytic approach consisted in the interpretation of transcriptional profiles of a single gene across multiple systems-scale profiling studies. The data from the different studies were not merged in a single unified meta-analysis. Thus it would be more appropriate to qualify this work as a “meta-interpretation”. It proved successful at identifying among a constellation of findings a common thread, the concomitant elevation of ADAM9 with conditions associated with extensive tissue damage. Concerns with regards to the quality of the public data used as input for meta-interpretation, for instance the introduction of uncontrolled confounding factors that may be technical (batch effects) or biological (demographics, treatment), should be mitigated by the fact that conclusions are based on data drawn from not one but multiple studies, and that these were vetted by institutional review boards and peer review. These mechanisms should ensure that only a minority of those studies would be affected by critical design or technical flaws. However, we also recognize that *in silico* cross-validation of seminal observations does not obviate the need for follow on studies or experimentation. Finally, the fact that the approach presented relies on interpretation of transcriptional profiles derived from a relatively large number of transcriptional studies presents another challenge given that the amount of background information that can be provided for each study cannot be exhaustive. The data browsing web application that we have used attempts to address this limitation by providing readers access to interactive figures that they can drill into to access detailed sample and study information. Taken together this study provides an original framework for the design of strategies aiming at leveraging vast amounts of high resolution molecular phenotyping data available in public repositories.

## Data availability

The data referenced by this article are under copyright with the following copyright statement: Copyright: © 2016 Rinchai D et al.

Data associated with the article are available under the terms of the Creative Commons Zero "No rights reserved" data waiver (CC0 1.0 Public domain dedication). 

All primary data presented in this manuscript can be accessed along with contextual information via the data browsing application described above and is also available in NCBI’s GEO public repository. GEO accession numbers (starting with GSE) are provided where appropriate throughout this manuscript along with the primary reference associated with the GEO record.

F1000Research: Dataset 1. Raw data of ADAM9 transcripts in blood in response to tissue damage, 10.5256/f1000research.6241.d138863 ^41^
