# Supplementary material for: Increased abundance of ADAM9 transcripts in the blood is associated with tissue damage
Source: F1000Res. 2016 Oct 24;4:89. Originally published 2015 Apr 9. [Version 2] doi: 10.12688/f1000research.6241.2 (PMC5130078; doi:10.12688/f1000research.6241.2)
Supplement: Peer review history

# Peer review: Referee response for version 2

**DOI:** 10.5256/f1000research.10567.r17151

**Published:** 2016-12-09

**Authors:** Adaikalavan Ramasamy

**Affiliations:** 1 The Jenner Institute, University of Oxford, Oxford, UK

The authors have done a thorough job of addressing the reviewers comment. The article is very easy to read now and presents a very useful application of their tool. Thank you.

I have read this submission. I believe that I have an appropriate level of expertise to confirm that it is of an acceptable scientific standard.

# Peer review: Referee response for version 2

**DOI:** 10.5256/f1000research.10567.r18034

**Published:** 2016-11-28

**Authors:** Andreas Ludwig

**Affiliations:** 1 Institute of Pharmacology and Toxicology, RWTH Aachen University Hospital, Aachen, Germany

Rinchai and co-workers nicely addressed all raised concerns. However, we still have two minor comments.

1. Figure 1 could be further improved. The presentation as scatter plot similar to the other figures as well as statistical analysis would strengthen the message and support the introduction to the readership.
2. Method section – statistical analyses: Please include “… and are indicated within the figure legends.”

I have read this submission. I believe that I have an appropriate level of expertise to confirm that it is of an acceptable scientific standard.

# Peer review: Referee response for version 2

**DOI:** 10.5256/f1000research.10567.r17152

**Published:** 2016-11-28

**Authors:** Caroline A. Owen

**Affiliations:** 1 Pulmonary Division, Brigham and Women's Hospital, Harvard Medical School, Boston, MA, USA

The authors have adequately responded to the concerns that were raised in the initial review. There is now a more comprehensive explanation of the study design and methods. The conclusions are balanced and justified.

I have read this submission. I believe that I have an appropriate level of expertise to confirm that it is of an acceptable scientific standard.

# Peer review: Referee response for version 1

**DOI:** 10.5256/f1000research.6696.r10054

**Published:** 2015-08-24

**Authors:** Caroline A. Owen

**Affiliations:** 1 Pulmonary Division, Brigham and Women's Hospital, Harvard Medical School, Boston, MA, USA

**The title and abstract of the manuscript:**

Both are appropriate. 

**The design, methods and analysis of the results from the study:**

The methods and design have been explained, and the analyses are appropriate for the topic being studied.  The results show impressive increases in ADAM9 gene expression in blood leukocytes in some disease states. However there are issues regarding the design/content of the study:

 

1. It is not clear from reading only the manuscript whether the controls and disease groups are matched with respect to age, sex, and/or race/ethnicity, and/or whether the disease groups studied have co-morbidities that might contributed to the differences in ADAM9 gene expression observed between the groups. It would be necessary to read many of the cited papers in order to obtain this information.
2. The microarray results do not appear to have been validated by performing real-time qPCR studies (for example) on any of the samples.
3. In the *in vitro *studies, details on the concentrations and incubation times for the agonists have not been provided in the methods, text or legends. It is possible that the concentrations and time points studied were not optimal for detecting increases in ADAM9 gene expression. 

**Data presentation:**

All of the results have been presented in the manuscript  However, in general more details about the experimental conditions in the figure legends would have been helpful to the reader.

**Discussion and conclusions:**

The discussion section could be expanded to include a discussion of the limitations of the study.  The discussion could have included a section on how the changes in ADAM9 gene expression detected in blood leukocytes might influence the pathogenesis of progression of the diseases that were studied based upon the known activities of this proteinase.

I have read this submission. I believe that I have an appropriate level of expertise to confirm that it is of an acceptable scientific standard, however I have significant reservations, as outlined above.

# Response

**Published:** 2016-10-11

**Authors:** Damien Chaussabel

**Affiliations:** Sidra Medical and Research Center, Qatar

We thank the reviewer for the valuable feedback and suggestions to improve our manuscript.

**The title and abstract of the manuscript:**

 Both are appropriate. 

**The design, methods and analysis of the results from the study:**

 The methods and design have been explained, and the analyses are appropriate for the topic being studied.  The results show impressive increases in ADAM9 gene expression in blood leukocytes in some disease states. However there are issues regarding the design/content of the study:

 1. It is not clear from reading only the manuscript whether the controls and disease groups are matched with respect to age, sex, and/or race/ethnicity, and/or whether the disease groups studied have co-morbidities that might contributed to the differences in ADAM9 gene expression observed between the groups. It would be necessary to read many of the cited papers in order to obtain this information.

***Authors:*** *This is a good point as such factors may indeed potentially confound analysis and undermine the conclusions advanced by the authors of those studies (since it would presumably affect not only ADAM9 but all the transcripts being measured).*

*Mechanisms exist that should help ensure that the study design and choice of selection of control subjects is appropriate at least in most studies: *

1. *One is IRB review that to some extent will evaluate the study design elements such as inclusion and exclusion criteria for case and control groups and will help ensure that results of the study will be meaningful and justify risk to the study population.*
2. *The second mechanism is peer review. Having conducted such studies ourselves and reviewed submissions of others concerns often come up regarding factors that might potentially confound analyses and that would need be addressed before publication.*

*In addition, the process of loading dataset and sample as well as study information in GXB as well as QC checks provide an additional opportunity to identify “faulty” designs.*

*These steps can of course only mitigate risk since even study investigators may not be aware of all the factors that could potentially confound the analysis. One of the potential advantages of the analytic strategy that we have employed is that it factors in results not from one but several studies carried out by different investigators in different geographic locations, often using different technology platforms. Thus the conclusions we derive from such “meta-interpretation” is likely to be rather robust with only a minority of the studies potentially being affected by study design.*

*It should also be noted that details concerning study design have been incorporated in GXB and therefore the reader does not have to access the original manuscript in order to locate the relevant information. Sample information is also available via GXB and can be accessed by 1) hovering of the mouse cursor over individual data points; 2) overlaying the information on the interactive bar graph; 3) accessing the table listing all available sample information.*

*We have acknowledged the point raised in the review and include some of the considerations outlined above in the conclusion of our manuscript:*

*“Concerns with regards to the quality of the public data used as input for meta-interpretation, for instance the introduction of uncontrolled confounding factors that may be technical (batch effects) or biological (demographics, treatment), should be mitigated by the fact that conclusions are based on findings from not one but multiple studies, and that all of them were vetted by institutional review boards and peer review. These mechanism should ensure that only a small minority of those studies would be affected by critical design or technical flaws.”*

 2. The microarray results do not appear to have been validated by performing real-time qPCR studies (for example) on any of the samples.

***Authors:*** *Confirmation by real-time PCR was not available for all studies, and when they were had not been performed for ADAM9 since it was not a focus of the systems-scale analyses. We did not have direct access to study samples and could not check levels of ADAM9 transcript ourselves. It should be noted that doing so would in any case only serve to validate the accuracy of the technology platform that the authors employed rather than the intrinsic value of ADAM9 as a biomarker. The approach that we employ provides a means for in silico validation of findings from an initial study across additional independent patient cohorts. However, we recognize that it does not ultimately obviate the need for follow on studies/experimentations.*

*We added the following sentence in the conclusion:*

*“We also recognize that such in silico cross-validation of our seminal observation does not obviate the need for follow on studies or experimentation.”*

 3. In the *in vitro *studies, details on the concentrations and incubation times for the agonists have not been provided in the methods, text or legends. It is possible that the concentrations and time points studied were not optimal for detecting increases in ADAM9 gene expression. 

**Data presentation:**

 All of the results have been presented in the manuscript. However, in general more details about the experimental conditions in the figure legends would have been helpful to the reader.

***Authors:*** *As requested by the reviewer* *we have added details in each dataset as shown in Figure legends through the manuscript. As mentioned above we have employed GXB as an interface between the readers and the papers that originally described the study and its findings. Information regarding study design or samples has been structured within GXB and can be accessed directly from the manuscript and in only a few clicks. It can also be represented graphically. We have also added a few sentences to highlight this point in the manuscript:*

*“Finally, the fact that the approach presented relies on interpretation of transcriptional profiles derived from a relatively large number of transcriptional studies presents another challenge given that the amount of background information that can be provided for each study cannot be exhaustive. The data browsing web application that we have used attempts to address this limitation by providing readers access to interactive Figures that they can drill into to access detailed sample and study information.”*

*Furthermore, we selected CXCL10 as a positive control to show that large levels of induction could be obtained for genes known to respond to those stimuli. Expression values for this gene range from nearly 10 up to nearly 40,000 units. And although this dataset is indeed publicly available we happen to have been the contributors (as is the case of a number of datasets being reanalyzed here) and had performed dose ranging and time course experiments prior to selection of the stimulation conditions.*

**Discussion and conclusions:**

 The discussion section could be expanded to include a discussion of the limitations of the study.  The discussion could have included a section on how the changes in ADAM9 gene expression detected in blood leukocytes might influence the pathogenesis of progression of the diseases that were studied based upon the known activities of this proteinase.

***Authors:*** *A new section has been added in the conclusion specifically to discuss limitations of the study (see additions mentioned above). We are yet unsure of the functional significance of elevation in levels of ADAM9, which on one hand may be beneficial to mediate tissue repair; on the other hand the fact that ADAM9 proteins or transcripts levels are found elevated in blood may be an indication of extensive tissue damage and be associated with poor outcome. Indeed we now for instance report in the context of GSE11375 (profiling of responses in the blood of trauma patient) that abundance of ADAM9 in patients who did not survive was significantly higher than those who survive. In another dataset GSE34205/GSE38900 (Viral infections) we now show that abundance of ADAM9 is correlated with degree of severity in pediatric viral infection (RSV, influenza and HRV infection), moreover level of ADAM9 transcript in patients who were ventilated were significantly higher than that who were non-ventilated. We have added these statements in the discussion.*

# Peer review: Referee response for version 1

**DOI:** 10.5256/f1000research.6696.r9961

**Published:** 2015-08-13

**Authors:** Andreas Ludwig, Daniela Dreymüller

**Affiliations:** 1 Institute of Pharmacology and Toxicology, RWTH Aachen University Hospital, Aachen, Germany

Rinchai and co-workers nicely present a re-analysis of existing genomic datasets, demonstrating an useful tool for quick establishment of functional hypotheses. By this, they suggest a novel function of ADAM9 as biomarker for tissue damage. The article is well written, but several concerns should be addressed before indexing.

1. Introduction: The authors give a nice review of the current literature. However, a link to their one study is missing. The introduction should include the motivation (“knowledge gap assessment”). Otherwise, readers could expect a detailed physiologic analysis of ADAM9 in tissue damage.
2. Methods: The description is very nice but could be adapted to journal style and shortened. The possibilities offered by the software could be summarized in a table.
3. Figure 1: The colour scheme in the figure and the legend are sorted differentially. In general, the figure is overloaded and the colour scheme not helpful. Differences were only observed for monocytes and neutrophils. These results should be included in figure 1, whereas the other results should be included as supplementary figure.
4. Supplementary Figure 2 to 5: I don’t see any additional information by this second plot type.
5. What is the difference between Suppl. Figure 2 4 and Figure 2?
6. Why are not all datasets mentioned in the text also shown and listed within the figures? This is very obvious for Figure 2.
7. The diagrams within the figures are very redundant especially due to the detailed description within the text. This space should be used to present more original data sets.
8. The tables should be summarized within one table. Further, the table should include all datasets analysed and mentioned in the text.
9. Figure 3: It would be helpful to mark the values for the different individuals, maybe by different colours to avoid the impression of a general outlier. Otherwise, changes after PAMP treatment could be possible.
10. Conclusion: To address the point of infection the authors include a stimulation of blood samples. However, this is not sufficient to draw the conclusion of a biomarker for tissue damage (also as a result of infection). Experiments with tissue cells, including scratch assays, stimulations with cytokines, and conditioned media from blood samples would provide further information and address the tissue damage effect in comparison to the infection effect.

We have read this submission. We believe that we have an appropriate level of expertise to confirm that it is of an acceptable scientific standard, however we have significant reservations, as outlined above.

# Response

**Published:** 2016-10-11

**Authors:** Damien Chaussabel

**Affiliations:** Sidra Medical and Research Center, Qatar

We thank the reviewers for their valuable feedback and suggestions to improve our manuscript.

 Rinchai and co-workers nicely present a re-analysis of existing genomic datasets, demonstrating an useful tool for quick establishment of functional hypotheses. By this, they suggest a novel function of ADAM9 as biomarker for tissue damage. The article is well written, but several concerns should be addressed before indexing.

 1. Introduction: The authors give a nice review of the current literature. However, a link to their one study is missing. The introduction should include the motivation (“knowledge gap assessment”). Otherwise, readers could expect a detailed physiologic analysis of ADAM9 in tissue damage.

***Authors:*** *As suggested we added a paragraph at the beginning of the introduction section to present the rationale behind the data mining approach that was employed. We experienced issues with one of our servers at some time. We checked the links and references provided in the introduction and it seems to be working fine now.*

 2. Methods: The description is very nice but could be adapted to journal style and shortened. The possibilities offered by the software could be summarized in a table.

***Authors:*** *We also took care of this. Since the description of the GXB tool has now been published and the code made openly available in Github we now point readers to these resources and shortened the paragraph describing the features of the software accordingly **(**Speake C, et al., J Transl Med 2015, Rinchai D, et al., F1000R 2016). A link to a tutorial video has also been added to the methods (https://www.youtube.com/playlist?list=PLtx3tvfIzJ9XkRKUz6ISEJpAhqKyuiCiD).*

*3. *Figure 1: The colour scheme in the figure and the legend are sorted differentially. In general, the figure is overloaded and the colour scheme not helpful. Differences were only observed for monocytes and neutrophils. These results should be included in figure 1, whereas the other results should be included as supplementary figure.

***Authors:*** *Thank you for pointing this out. In the original version of the Figure we used the graphic exported directly with GXB. However, we agree that it is difficult to read, especially without interactive features that allow overlay of sample information, sorting and mouse overs. Another reviewer also suggested retaining only the neutrophil and monocyte data the plot for Figure 1 and we have made these changes accordingly.*

 4. Supplementary Figure 2 to 5: I don’t see any additional information by this second plot type.

***Authors:*** *Supplementary Figures 2-5 represent the data exactly how they can be visualized interactively in the GXB. We felt this might be helpful given the fact that we provide links throughout the manuscript that lead to interactive version of these plots. We are now providing this rationale in the legend of the supplementary figures.*

 5. What is the difference between Suppl. Figure 2 4 and Figure 2?

***Authors:*** *Same rationale as stated above, we used Supplementary Figures representing the original data exactly how they can be visualized interactively in the GXB. The links to the interface of each graph are provided in legends of each Supplementary Figures.*

 6. Why are not all datasets mentioned in the text also shown and listed within the figures? This is very obvious for Figure 2.

***Authors:*** *We in fact initially tried to show the results from all of the studies. But since some of the Figures make reference to a rather large number of datasets and that we are able to provide links to interactive graphs we decided to only select a subset of the key studies that best support the points that we were making. In response to the reviewers' comments we are now listing all the studies in the Figure legend and accompanying Table. Readers can access the data for each study by clicking the associated hyperlinks. All the studies mentioned in the text are also represented on the graphical abstract.*

 7. **The diagrams within the figures are very redundant especially due to the detailed description within the text. This space should be used to present more original data sets.

***Authors:*** *We did not properly communicate the purpose of these diagrams that constitute graphical legend and allow presentation of the data in a semi-structured format that is both human and machine readable. We are now providing a rationale (see below) and have re-positioned them at the bottom of the Figure, which will hopefully work better.*

*Rationale: “Diagrams have been incorporated within each Figure. These have a dual purpose, first they provide readers with a graphical summary of the findings and second constitute an attempt a structuring information for future computational applications. Indeed, an important limitation of communicating biomedical knowledge in the form of research articles is that it consists in unstructured information (free text). This type of information is notoriously difficult to extract by computational means [e.g.* *Chaussabel D. Am J Pharmacogenomics 2004; 4: 383-93* *].* *Standardized graphical summaries such as the ones provided in this manuscript constitutes structured information that is both human readable and computationally tractable. The need for such solutions will become more pressing as the biomedical literature continues to grow exponentially to such scales that it can only be very narrowly apprehended by research investigators.”*

 8 *. *The tables should be summarized within one table. Further, the table should include all datasets analysed and mentioned in the text.

***Authors:*** *We thanks to reviewer for raising this point. As mentioned earlier we have added studies that have been previously omitted. But as far as merging all datasets in one table we were concerned after making an attempt that it would be difficult for reader to track down information about a given dataset if the list is too extensive. Also we reverted to the original format where separate tables are linked to each individual figure.*

 9. Figure 3: It would be helpful to mark the values for the different individuals, maybe by different colours to avoid the impression of a general outlier. Otherwise, changes after PAMP treatment could be possible.

***Authors:*** *As suggested by reviewers, we labeled the value of different individual by using different colors in the PAMPs treatment dataset (GSE30101). We found that ADAM9 levels didn’t show significant outlier response, with the exception of the green subject that shows low response to HKSA in comparison to the other subjects. This could be explained by donor-specific variation in the subject’s ability to respond. Overall the magnitude of response to such stimuli remains especially low, especially when compared to CXCL10 which served as a positive control and did not reach significance. Donor information was not available for GSE32862*

 10. Conclusion: To address the point of infection the authors include a stimulation of blood samples. However, this is not sufficient to draw the conclusion of a biomarker for tissue damage (also as a result of infection). Experiments with tissue cells, including scratch assays, stimulations with cytokines, and conditioned media from blood samples would provide further information and address the tissue damage effect in comparison to the infection effect.

***Authors:*** *Indeed we are reporting an association and direct evidence will have to be obtained experimentally. We have changed the title to better reflect this fact, and it now reads: “ Increased abundance of ADAM9 transcripts in the blood is associated with tissue damage”.*

*Confirmation experiments will require the analysis of large number of sample for validation and will require further investigation, we are working to secure necessary funding and ethics approval for follow on studies, which is taking more time than anticipated. Some datasets available in GEO include experiments that are relevant, this is for instance the case GSE30101 where whole blood that was stimulated with microbe-derived as well as host-derived factors such as pathogen-associated molecular patterns (PAMPs), inflammatory cytokines (IL18, TNF), as well as interferon type I and Type II). Prompted by your comment we also have checked expression of ADAM9 transcript in additional datasets. Transcript abundance were for instance measured in the lungs of C57BL/6 in a model of lung inflammation and injury ( GSE2411) [* *Kennedy-Crispin M. et al., J Invest Dermatol 2012* *]. The result showed that the abundance of ADAM9 was significantly higher in mice that developed acute lung injury after exposure to low-dose LPS and mechanical ventilation ( GSE2411). In an experimental model of epidermal injury ( GSE30355) the abundance of ADAM9 was significantly higher in injured epidermis (sorted keratinocyte (KC)) as comparison to uninjured condition (laser capture microscopy or in vitro cultured keratinocytes) [* *Altemeier WA. et al., J Immunol 2005* *]* *. Additionally, in a murine dermal burn wound model ADAM9 transcript of mouse thermal injury induced increased over the time at 0, 2 hours, 3 days respectively ( GSE460).*

*We have added a description of these findings in the result section under the paragraph entitled “* *The abundance of ADAM9 increases following tissue injury and sterile inflammation* *” and are also including a new Supplementary Figure 6.*

# Peer review: Referee response for version 1

**DOI:** 10.5256/f1000research.6696.r8789

**Published:** 2015-07-16

**Authors:** Adaikalavan Ramasamy

**Affiliations:** 1 The Jenner Institute, University of Oxford, Oxford, UK

Rinchai *et al. *suggest a novel role for ADAM9 by mining exisiting dataset. This clever re-use of existing dataset is a demonstration on how scientists can test new hypothesis quickly, inexpensively and with more robustness. They also provide a web tool based on 172 curated datasets ( https://gxb.benaroyaresearch.org/dm3/geneBrowser) which makes is a practical resource.

All sections of the article is extremely well written and I strongly recommend the article be indexed subject to the following comments.

1. **Introduction:** The introduction starts with the Refseq definition of ADAM9 and a thorough review of existing literature on gene function of ADAM9. It left me wondering what motivated them to ADAM9 until the first section of Results (Knowledge gap assessment). It would be useful to the reader if a brief sentence or two on the motivation to study this gene was at the beginning of the Introduction section.
2. **Figure 1:** I find it very difficult to color match the Cell type on the x-axis of Figure 1 especially when it appears legend colors are sorted differently. A plot with seven smaller panels (one for each cell type) or even just 2 panels (nueturophils and monocytes) might be clearer. Can you add GSE60424 to title of Figure 1?
3. General comment on **Figures 2 - 5 ** and **Tables 1 - 4**:
  a) There is an inconsistency in the number of datasets stated in text and demonstrated in the figure. E.g. the text for Figure 2 talks about seven datasets but figure only shows three and Table 1 also talks about three datasets but includes SOJIA vs Control and HIV vs Control from GSE29536.
  b) I find the process diagrams (top half of figures) distracting and redundant with text and legend. This space could be used to incorporate the other studies. I suggest incorporating the cell type and measurement type after the study names on plot (e.g. GSE34205 \n microarray on whole blood; GSE29536 \n RNA-seq on neutrophils). Legend is well described.
  c) The column for "Avg A - Avg B" is meaningless especially when comparing different platforms. The fold change (Avg A / Avg B) is more meaningful and would be worth stating to two decimal points. 
  d) If possible, combine Tables 1 - 4 into one page, possibly a large table with subheadings for during infection, after treatment with PAMPs, during tissue remodelling etc ... T
4. **Forest plot:** An alternative/additional suggestion to 3d is to present the data visually in the form of a forest plot with subheadings (e.g. https://www.nichd.nih.gov/cochrane_data/mcguirew_13/fig2019799225306621155.png; test of heterogeneity might not be necessary). This allows the readers to visually scan all of the in one page. Many ways of doing this but I suggest calculating the Glass effect size (see R codes below) followed by foresplot function from R package rmeta or forestplot. 
  The authors might also find such a plot on their webtool useful in the long run but this is beyond the scope of current paper.
  effectSize <- function(baseline, posttest){
   ## Source http://www.meta-analysis.com/downloads/Meta-analysis%20Effect%20sizes%20based%20on%20means.pdf
  stopifnot( length(posttest)==length(baseline) ) ## assume the data is in same patient order
  w <- which( !is.na(posttest) & !is.na(baseline) )
  posttest <- posttest[w]
  baseline  <- baseline[w]
    r      <- cor( posttest, baseline )
   diff   <- posttest - baseline
   n      <- length(diff)
   S.diff   <- sqrt( var(baseline) + var(posttest) - 2*cov(baseline, posttest) )
     # note: var(x - y) = var(x) + var(y) - 2cov(x, y)
    S.within <- S.diff/sqrt(2*(1-r))      # same as S.pooled
    ## Cohen's d ##
   d     <- mean(diff)/S.within
   var.d <- ( 1/n + (d^2)/(2*n) ) * 2 * (1-r)
   ## Hedge's g estimate ##
    cf    <- 1 - 3/(4*n -5)
    g     <- cf*d
    var.g <- cf^2 * var.d
    se.g  <- sqrt(var.g)
   rm(posttest, baseline, w, r, diff, n, S.diff, S.within, d, var.d, cf, var.g)
   return( c( g=g, se.g=se.g, LCL=( g - 1.96*se.g), UCL=( g + 1.96*se.g) ) )
  }
5. I am unclear what Supplementary Figures 2, 3, 4 and 5 adds to the paper.
6. There is emerging evidence that the monocyte to lymphocyte ratio has relevance to susceptibility to infectious diseases (e.g. Wang *et al.*, 2015; Naranbhai *et al.*, 2014; Warimwe *et al.*, 2013). Could you speculate/demonstrate how you could potentially use your resource to test for this hypothesis? Perhaps using cell deconvulation methods on whole blood?

I have read this submission. I believe that I have an appropriate level of expertise to confirm that it is of an acceptable scientific standard, however I have significant reservations, as outlined above.

# Response

**Published:** 2016-10-11

**Authors:** Damien Chaussabel

**Affiliations:** Sidra Medical and Research Center, Qatar

We would like to thank reviewer for kindly comments and suggestions to improve our manuscript.

 Rinchai *et al. *suggest a novel role for ADAM9 by mining exisiting dataset. This clever re-use of existing dataset is a demonstration on how scientists can test new hypothesis quickly, inexpensively and with more robustness. They also provide a web tool based on 172 curated datasets ( https://gxb.benaroyaresearch.org/dm3/geneBrowser) which makes is a practical resource.

 All sections of the article is extremely well written and I strongly recommend the article be indexed subject to the following comments.

 1. **Introduction:** The introduction starts with the Refseq definition of ADAM9 and a thorough review of existing literature on gene function of ADAM9. It left me wondering what motivated them to ADAM9 until the first section of Results (Knowledge gap assessment). It would be useful to the reader if a brief sentence or two on the motivation to study this gene was at the beginning of the Introduction section.

***Authors:*** *Thank you for raising this point, we agree that it would be better to start with such a description. So we have now added a paragraph explaining the “Collective data to knowledge” approach as the first paragraph of the introduction section.*

 2. **Figure 1:** I find it very difficult to color match the Cell type on the x-axis of Figure 1 especially when it appears legend colors are sorted differently. A plot with seven smaller panels (one for each cell type) or even just 2 panels (neutrophils and monocytes) might be clearer. Can you add GSE60424 to title of Figure 1?

***Authors:*** *We agree with this suggestion. We initially wanted to use the plot as it would appear to the reader when accessing the GXB via the link provided, but it is rather difficult to interpret without the interactive features built into the software tool (overlay of sample information, sample sorting, pop ups etc…). Also per your and another reviewer’s suggestion we changed the plot of Figure 1 showing only neutrophil and monocyte data.*

 3. General comment on **Figures 2 - 5 **and **Tables 1 - 4**:

 a) There is an inconsistency in the number of datasets stated in text and demonstrated in the figure. E.g. the text for Figure 2 talks about seven datasets but figure only shows three and Table 1 also talks about three datasets but includes SOJIA vs Control and HIV vs Control from GSE29536.

***Authors:*** *Thanks for pointing this out. Not all dataset analyzed were plotted on the figures but all are now listed in the tables, in the textual figure legends, are represented on the graphical figure legends and can be accessed via the links provided.*

 b) I find the process diagrams (top half of figures) distracting and redundant with text and legend. This space could be used to incorporate the other studies. I suggest incorporating the cell type and measurement type after the study names on plot (e.g. GSE34205 \n microarray on whole blood; GSE29536 \n RNA-seq on neutrophils). Legend is well described.

***Authors:*** *This point has been raised by another reviewer as well and is obviously important. We did not properly communicate the purpose of these diagrams which are meant as “graphical figure legends”. We aimed at structuring the information communicated and also help readers navigate the many finding that are reported while providing links to interactive figures and make details regarding study design more readily accessible (which a third reviewer deemed particularly important). In addition to providing the rationale for including those graphical figure legends we also moved them at the bottom of each figure, which is really the most logical spot for them to be.*

*Rationale: Diagrams have been incorporated within each figure. These have dual purpose, first providing readers with a graphical summary of the findings and second constitute an attempt a structuring information for future computational applications. Indeed, an important limitation of communicating biomedical knowledge in the form of research articles is that it consists in unstructured information (free text). This type of information is notoriously difficult to extract by computational means [* *Chaussabel D. Am J Pharmacogenomics 2004; 4: 383-93* *]. Standardized graphical summaries such as the ones provided in this manuscript constitutes structured information that is both human readable and computationally tractable. The need for such solutions will become more pressing as the biomedical literature continues to grow exponentially to such scales that it can only be very narrowly apprehended by research investigators.*

 c) The column for "Avg A - Avg B" is meaningless especially when comparing different platforms. The fold change (Avg A / Avg B) is more meaningful and would be worth stating to two decimal points.

***Authors:*** *We agree that it indeed cannot be compared across platforms, which we did not intend to do since rather than a meta-analysis our approach consists in a “meta-interpretation” across publicly available datasets. However, it is a good indication of robustness of the changes that are measured. We have used this criterion for many years to weed out genes that show high fold change but for expression levels that are close to background levels, which we have found to be poorly reproducible. For example, in case where fold change = 3 difference if A=30 and B=10 will be 20 which might be about twice the background intensity of the chip; whereas if A=300 and B=100, A/B is still = 3 but A-B is 200 or twenty time the background intensity. So having this information can help decide whether the changes that are observed are likely to be robust.*

 d) If possible, combine Tables 1 - 4 into one page, possibly a large table with subheadings for during infection, after treatment with PAMPs, during tissue remodelling etc ... T

***Authors:*** *Thank you for raising this point, we initially considered this possibility but found that It would be too much information in one table.  We were also concerned that it would make it too difficult for the reader to locate this information.*

 4 *.* **Forest plot:** An alternative/additional suggestion to 3d is to present the data visually in the form of a forest plot with subheadings (e.g. https://www.nichd.nih.gov/cochrane_data/mcguirew_13/fig2019799225306621155.png; test of heterogeneity might not be necessary). This allows the readers to visually scan all of the in one page. Many ways of doing this but I suggest calculating the Glass effect size (see R codes below) followed by foresplot function from R package rmeta or forestplot. 

 The authors might also find such a plot on their webtool useful in the long run but this is beyond the scope of current paper.

 effectSize <- function(baseline, posttest){

  ## Source http://www.meta-analysis.com/downloads/Meta-analysis%20Effect%20sizes%20based%20on%20means.pdf

 stopifnot( length(posttest)==length(baseline) ) ## assume the data is in same patient order

 w <- which( !is.na(posttest) & !is.na(baseline) )

 posttest <- posttest[w]

 baseline  <- baseline[w]

   r      <- cor( posttest, baseline )

  diff   <- posttest - baseline

  n      <- length(diff)

  S.diff   <- sqrt( var(baseline) + var(posttest) - 2*cov(baseline, posttest) )

    # note: var(x - y) = var(x) + var(y) - 2cov(x, y)

   S.within <- S.diff/sqrt(2*(1-r))      # same as S.pooled

   ## Cohen's d ##

  d     <- mean(diff)/S.within

  var.d <- ( 1/n + (d^2)/(2*n) ) * 2 * (1-r)

  ## Hedge's g estimate ##

   cf    <- 1 - 3/(4*n -5)

   g     <- cf*d

   var.g <- cf^2 * var.d

   se.g  <- sqrt(var.g)

  rm(posttest, baseline, w, r, diff, n, S.diff, S.within, d, var.d, cf, var.g)

  return( c( g=g, se.g=se.g, LCL=( g - 1.96*se.g), UCL=( g + 1.96*se.g) ) )

 }

***Authors:*** *We thanks reviewer for this valuable suggestion and sharing the R code! Our GXB tool is still in development and we hope to add additional options for data visualizations in the future so this is perfect.*

 5. I am unclear what Supplementary Figures 2, 3, 4 and 5 adds to the paper.

***Authors:*** *We intended to show in Supplement Figures 2-5 the data exactly how they can be visualized interactively in the GXB. We could remove these figures but since this information is merely added as a supplement we felt that there is no harm in leaving it as is.*

 6. There is emerging evidence that the monocyte to lymphocyte ratio has relevance to susceptibility to infectious diseases (e.g. Wang *et al.*, 2015; Naranbhai *et al.*, 2014; Warimwe *et al.*, 2013). Could you speculate/demonstrate how you could potentially use your resource to test for this hypothesis? Perhaps using cell deconvulation methods on whole blood?

***Authors:*** *This is a very interesting hypothesis but perhaps beyond the scope of this manuscript; it might be possible to identify relevant dataset but such outcome measure is not easily ascertained at least in humans (i.e. susceptibility to infection)*
